# Fundamental properties of high-quality carbon nanofoam: from low to high density

**DOI:** 10.3762/bjnano.7.197

**Published:** 2016-12-27

**Authors:** Natalie Frese, Shelby Taylor Mitchell, Christof Neumann, Amanda Bowers, Armin Gölzhäuser, Klaus Sattler

**Affiliations:** 1 Department of Physics and Astronomy, University of Hawaii, 2505 Correa Road, Honolulu, HI 96822, USA; 2 Physics of Supramolecular Systems and Surfaces, Bielefeld University, Universitätsstraße 25, 33615 Bielefeld, Germany

**Keywords:** carbon nanofoam, helium ion microscopy, hydrothermal carbonization, nanocarbons

## Abstract

Highly uniform samples of carbon nanofoam from hydrothermal sucrose carbonization were studied by helium ion microscopy (HIM), X-ray photoelectron spectroscopy (XPS), and Raman spectroscopy. Foams with different densities were produced by changing the process temperature in the autoclave reactor. This work illustrates how the geometrical structure, electron core levels, and the vibrational signatures change when the density of the foams is varied. We find that the low-density foams have very uniform structure consisting of micropearls with ≈2–3 μm average diameter. Higher density foams contain larger-sized micropearls (≈6–9 μm diameter) which often coalesced to form nonspherical μm-sized units. Both, low- and high-density foams are comprised of predominantly sp^2^-type carbon. The higher density foams, however, show an advanced graphitization degree and a stronger sp^3^-type electronic contribution, related to the inclusion of sp^3^ connections in their surface network.

## Introduction

Nanofoams are of considerable current interest due to their unique structure, which lies between two and three dimensions, allowing for many new types of materials with promising new functions for future technologies. Since many material functions rely on molecule–surface interactions and low-dimensional properties, materials with large surface areas and a quantum-confined nanoscale nature are of particular interest. Such conditions are provided by porous materials with nanometer dimensions. The main questions to be answered are related to the synthetic methods for formation, the foam morphologies, and the electronic and vibronic properties.

Nanofoam materials derived from various chemical elements have been investigated. In particular, foams from transition metals and noble metals have attracted attention, and interesting applications have been developed. Nickel nanofoam has been used for glucose sensing [1] and also been suggested for high-performance supercapacitor electrodes [2]. Copper nanofoams containing both micropores and nanopores have been produced for potential use in energy applications [3], and furthermore, copper nanofoam substrates were used for the production of electrocatalysts [4]. Gold nanofoams have been found to show excellent catalytic properties [5]. Hydrogen thermoporometry was used to determine the pore architecture of gold and titania nanofoams [6]. Nanofoams from low-mass elements are interesting with respect to possible energy harvesting applications, e.g., high hydrogen storage capacity of 10 wt % in Be nanofoam has been predicted [7]. Besides cellular structures, other scaffolds have been suggested in some cases, for example, for gold. Foams of connected gold nanowires have been designed in molecular dynamics computer simulations [8]. Also, for materials such as glass compounds, nanofoam with a three-dimensional scaffold of interconnected nanowires have been produced [9]. A few studies of semiconductor and insulator nanofoams have also been reported. A sol–gel process was used to produce silicon nanofoam and acousto-optic characteristics were probed [10]. Silica nanofoam was used as a support material for highly active single- and dual-site catalysts [11]. Macroporous structured materials with cavities of about 100 nm in diameter have been reported for silica [12], and polyhedral nanofoam shells with open cells have been produced [13]. A labyrinth internal structure has been found for tantalum oxide nanofoams [14]. All these studies show that functional nanofoam materials can be produced from various chemical elements and compounds and that these foams are promising materials for future technologies. However, many questions about the relationship between foam morphologies and corresponding formation parameters are not answered. This is in particular true for nanofoams of carbon, which can occur in many different structural forms.

Among the chemical elements, carbon has the largest variety of possible electronic configurations. Therefore, many researchers have focused their interest in the study of novel carbon structures. A variety of production techniques have resulted in a large number of carbon materials with different sizes and structural properties. In particular, nanocarbons have been the focus since their properties depend critically on the synthetic methods, and as a consequence, many exciting developments have been reported [15].

The unique ability of carbon to occur in the form of sp^1^, sp^2^, and sp^3^ hybridizations and combinations between these configurations results in a wide range of morphologies. Among these are the complex carbon materials with nanometer-sized porous structures such as carbon aerogels [16] and carbon sponges [17]. Carbon nanofoams have first been produced using pulsed laser ablation of glassy carbon in argon atmosphere [18] and later, as graphite in liquid nitrogen [19]. Pulsed-laser deposition has also been used for the fabrication of carbon nanofoam electrodes [20]. Carbon nanotube foam in the form of aligned layered carbon nanotube structures infiltrated with pyrolytic carbon is considered useful in water treatment and oil spill cleanup [21]. Carbon nanofoam paper [22] is proposed for energy storage applications. Carbon nanofoam composites may be used for electrochemical capacitor electrodes [23] with potential application in high energy density electrochemical supercapacitors.

A promising formation method for high-quality carbon nanofoam is the low-temperature hydrothermal carbonization of sucrose [24]. In order to produce advanced carbon nanomaterials, there is a need for further development of this method since it is environmentally friendly without the use of toxic chemicals. Using this technique, the foams tend to be composed of micrometer-sized spheres of predominantly sp^2^ carbon that forms a three-dimensional open scaffold. These so-called micropearls are usually detected as individual units which are weakly connected, forming the foam structure. Their interaction is not strong enough for coalescence to occur. The hydrothermal process allows for the variation of growth parameters which may lead to further foam morphologies. The study of the parameter–morphology relationship can help to better understand the hydrothermal carbonization process, and in addition, to tune the growth toward particular materials structures. Such studies can considerably expand the possibility of production of nanomaterials by hydrothermal carbonization.

In this paper, we probe two carbon nanofoam materials with different densities produced by naphthalene-assisted hydrothermal sucrose carbonization. Structural, compositional, and vibrational information is obtained by helium ion microscopy (HIM), XPS (X-ray photoelectron spectroscopy), and Raman spectroscopy, respectively. We find significant differences between low- and high-density foams. The low-density foams consist of small, individual micropearls with only a few being interconnected. The high-density foams are formed by much larger micropearls where most of them are coalesced into larger units. Both the signatures in the XPS and the Raman spectra are different for the two types of foams. We discuss the foams assuming a 3D-graphene-type network with open cavities forming the micropearl structures.

## Experimental

### Hydrothermal reactor

Two stainless steel autoclaves, each with a volume of 130 mL, were used for these studies; a setup which is similar to that applied in our previous studies on carbon nanofoam [24]. The autoclaves were filled with 0.5 M sucrose solution, and subsequently 2 mg of naphthalene was added to each container. Then, the autoclaves were sealed and connected to the heating supply. Subsequently, one of the autoclaves was kept at 150 °C, the other at 185 °C, each for 48 h. After cooling and opening, the carbon foams which had formed in the container were extracted. Then, boiling water was applied to separate the solid carbon foam from the remaining liquid solution. After drying, the mass densities of the foams were determined using a predefined volume container and a high-precision balance.

### Helium-ion microscopy

An Orion Plus (Carl Zeiss) helium-ion instrument was used. An acceleration voltage of 34.9 keV and a beam current of 0.6 pA were applied, and secondary electrons were detected to obtain the images. Since the samples were not coated with conductive layers, an electron flood gun was applied to stabilize charging. Prior to imaging, the foam material was attached to the HIM sample holder with conductive carbon pads.

The HIM induces a high brightness, low-energy spread, subnanometer-size beam of helium ions [25]. An atomic-level ion source (ALIS) is used for the production of the helium ions. In this source, the beam is formed by ionization of helium atoms at a sharp needle held at high positive voltage. Such a beam of He ions has a diameter of typically less than one atom. It is focused onto the substrate under investigation using an electro-optical lens system. The image is then provided either by ionoluminescence [26], Rutherford backscattering of the ions [27], or secondary electron emission [28]. The high resolution is given by the small subsurface ion beam spread [29]. The instrument is suitable for the imaging of low-mass elements such as carbon due to its very high brightness. In addition to imaging, elemental analysis can be achieved with the He-ion microscope [30]. In this work we chose to use the secondary electron emission setup for imaging of our samples.

### Raman spectroscopy

Raman spectroscopy is a valuable method for the characterization of carbon nanofoam [24]. In these studies, Raman spectra were recorded using a micro Raman spectrometer (LabRAM ARAMIS) which was operated in the backscattering mode. As light source we used a diode laser at 473 nm, which was focused onto the sample with a 10x microscope objective. A thermoelectrically-cooled CCD detector was applied to detect the scattered photon intensity. For the measurement, the foam was mounted on conductive carbon tape.

### X-ray photoelectron spectroscopy (XPS)

X-ray photoelectron spectroscopy was performed in a UHV system (Multiprobe, Omicron) at a pressure <10^−9^ mbar, as applied in our previous work on carbon nanofoams [24]. The combination of monochromatic Al Kα irradiation, an electron analyzer (Sphera) with a resolution of 0.9 eV, and an emission angle of 20° was used. The deconvolution of XPS peaks was performed by consideration of a Shirley background and symmetric Voigt functions. The foam was attached to the XPS sample holder by conductive carbon tape.

In XPS, the elemental composition is obtained by measuring the binding energy of photoelectrons which are emitted when a material is irradiated by X-rays. The method is surface sensitive due to the small inelastic mean free path of the photoelectrons. With XPS, bond information can also be obtained since the core level energies are affected by the chemical environment of the atoms. For example, for carbon atoms, bonds such as C–C, C=C, C–O, and C=O can be distinguished.

A large variety of carbon materials have been investigated by XPS. In particular, XPS reveals important bonding information about carbons with nanoscale units such as carbon nanotubes [31], diamond-like carbon films [32], nanostructured carbon films [33], tetrahedral amorphous carbon films [34], amorphous carbon [35], nanoporous carbon [36], activated carbon [37], or carbon black [38]. In addition, XPS is important for the analysis of chemical structure [39], in particular, for the investigation of the sp^2^/sp^3^ hybridization ratio [35].

## Results

### Density

We determined the density of the foam by averaging over the densities of several foam samples. Using two different process temperatures of 160 and 185 °C in the autoclave resulted in average densities of 0.104 g·cm^−3^ and 0.278 g·cm^−3^, respectively. These densities are significantly lower than the densities of graphite (2.267 g·cm^−3^), amorphous carbon (1.8–2.1 g·cm^−3^), carbon nanotubes (1.6 g cm^−3^) or diamond (3.515 g·cm^−3^) [40].

Various types of low-weight carbons have been reported in the literature, with densities typically between 100 and 300 mg·cm^−3^. Among these are carbon aerogels [41–43], amorphous carbon nanoparticles [44–45], nanoporous carbons [46], carbon nanotube scaffolds [47], and carbon foams [48–49]. The densities of these carbon materials are significantly lower compared to “heavy carbons” such as pristine graphite (2.26 g·cm^−3^), CVD grown carbon films (2.14 g·cm^−3^ [50]), or carbon nanotube forests (1.6 to 0.38 g·cm^−3^ [51]). Some techniques have been reported which allow the production of carbons in a wide density range (0.20 to 1.4 g·cm^−3^ [52]) when production parameters were varied.

### Microscopy

**Figure 1:**
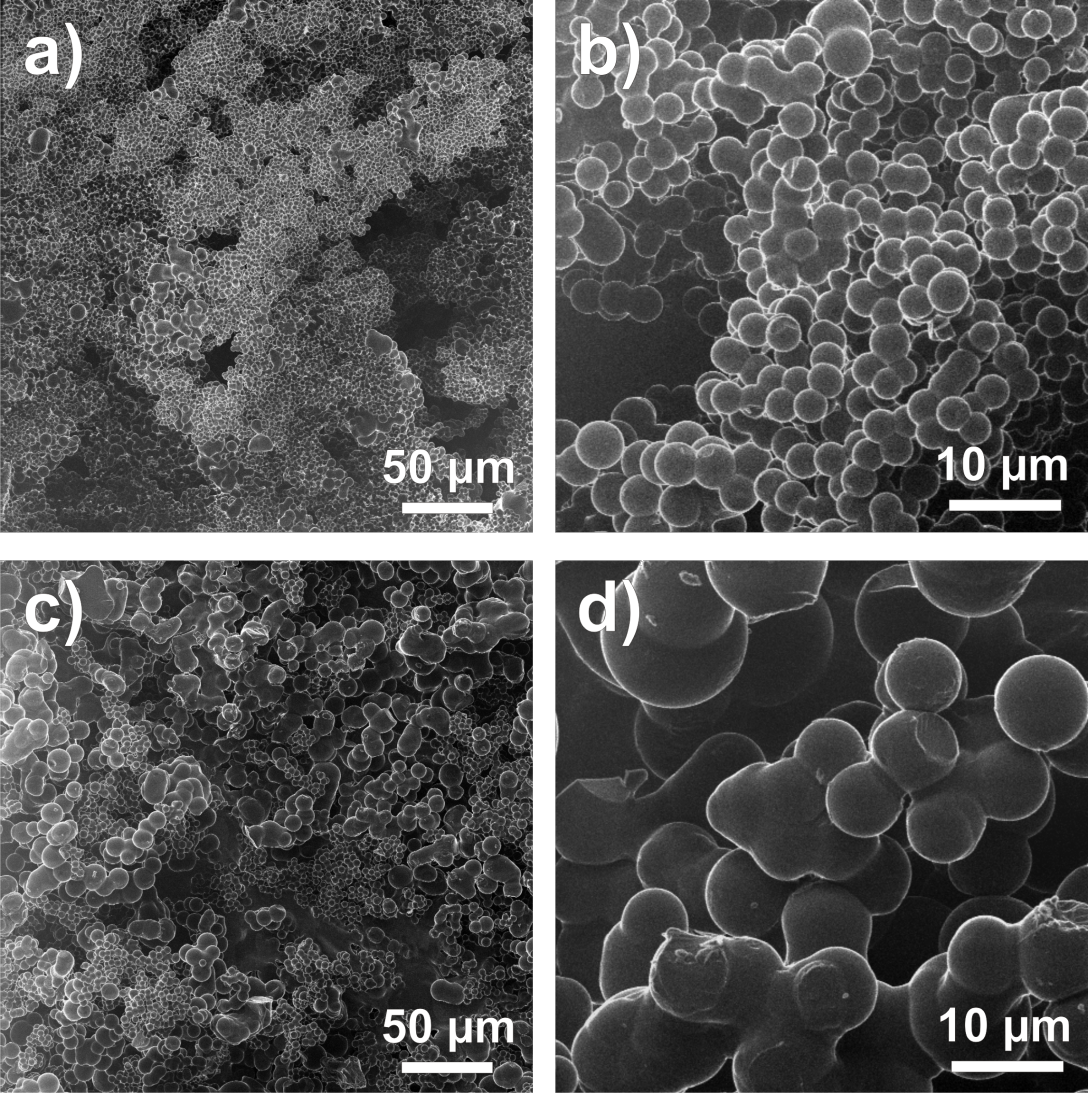
Helium-ion microscopy (HIM) images of low-density (a,b) and high-density (c,d) carbon nanofoams, with different magnifications.

Figure 1a,b shows HIM images of low-density (LD) nanofoam, and high-density (HD) nanofoams in the lower-panels (c,d). In Figure 1a,c, relatively large-scale images are shown, with a scale bar of 50 µm, for both, (LD) (a) and HD (c). It can be seen that both foams have a granular structure composed of small spherical-like units. This is further observed in the higher-magnified images of Figure 1b,d, with a scale bar of 10 µm. The low-density foams consist of nearly monodisperse small spheres with diameters between 2 and 3 µm. These micropearls have a narrow size distribution. The sample morphology is quite uniform over large areas. Occasionally, the micropearls are grown together forming larger units. However, in most cases, the micropearls are individually separated from each other. This is different for the high-density foams, displayed in Figure 1c,d. Here, the individual carbon spheres are larger in size and show a strong tendency for coalescence. In particular, Figure 1d shows that the spheres are connected with necks formed between them. Obviously the two materials, with low or high densities, are significantly different in form and size as seen by comparison of the images in Figure 1b,d.

### Raman spectroscopy

**Figure 2:**
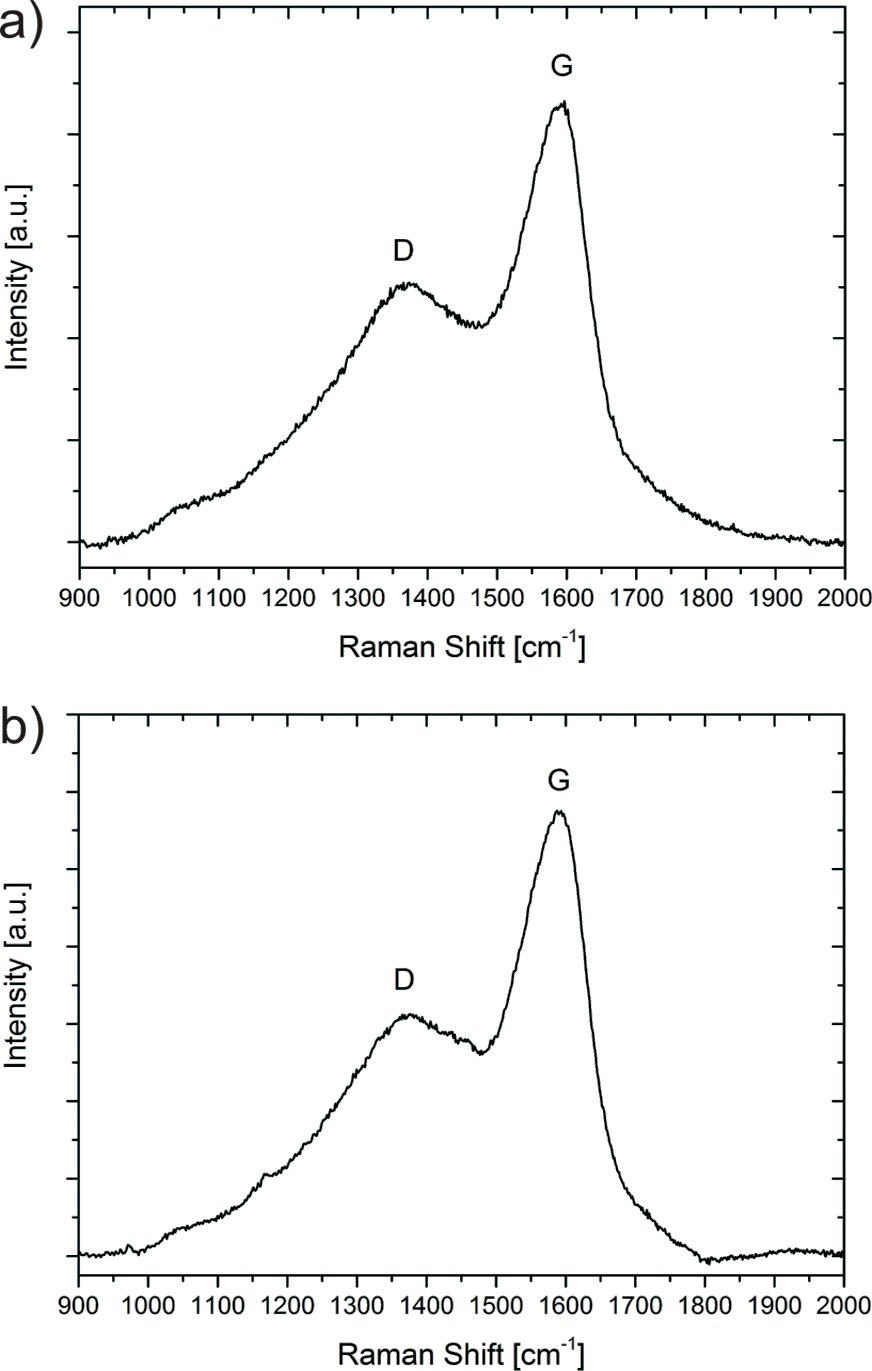
Raman spectra in the 900–2000 cm^−1^ range, for low- and high-density carbon nanofoams.

The Raman spectra for low-density and high-density nanofoams are shown in Figure 2a,b, respectively. The two prominent carbon peaks are displayed. For the low-density foams we find the G-peak at 1890 cm^−1^ and the D-peak at 1378 cm^−1^. Both peaks are slightly shifted to lower wavenumbers of 1587.04 cm^−1^ and 1376.63 cm^−1^ in the high-density foam, respectively (Figure 2b). This shift may be explained by the additional strain of the sp^2^ network due to the formation of the neck area between spheres. In addition, the ratio of these peaks is different for the two types of foams. In the high-density foam, the D- to G-peak ratio is 0.54, slightly smaller than the value of 0.59 as determined for the low-density foam. Even though the difference in the ratio is small, it suggests that the high-density foam is of an increased graphitization stage, having a higher content of sp^2^-type carbons with extended ordered graphitic regions. Increased process temperatures, resulting in higher-density foams, obviously assists the formation of ordered graphitic wall structures. On the other hand, it also leads to extended growth of the micropearls to larger sizes and to the coalescence of the spheres.

In disordered carbon, the G- and D-peaks are usually grown together forming one broad peak. In this respect it is interesting to note that the G- and D-distributions for the nanofoams in this study are formed by distinct peaks. This indicates that the micropearls consist of a relatively well-organized internal graphitic carbon structure.

We note that there is a gradual increase in the Raman intensity from 900 to 1376 cm^−1^. This may be due to unidentified vibrations of various types of nanocarbons and possibly of hydrocarbons adsorbed in the foams. We note that at 1180 cm^−1^, a peak was determined for nanocrystalline diamond films [53]. Also, two Raman features at 1180 and 1490 cm^−1^ in addition to the G and D peaks were observed for carbon films prepared by magnetron sputtering [53]. A correlation between the 1180 cm^−1^ peak and the sp^3^ content in these films was found [53]. At approximately this wavenumber, we see a small feature in the spectrum of Figure 2b. This indicates that a small diamond-like contribution is present in these foams.

### XPS

**Figure 3:**
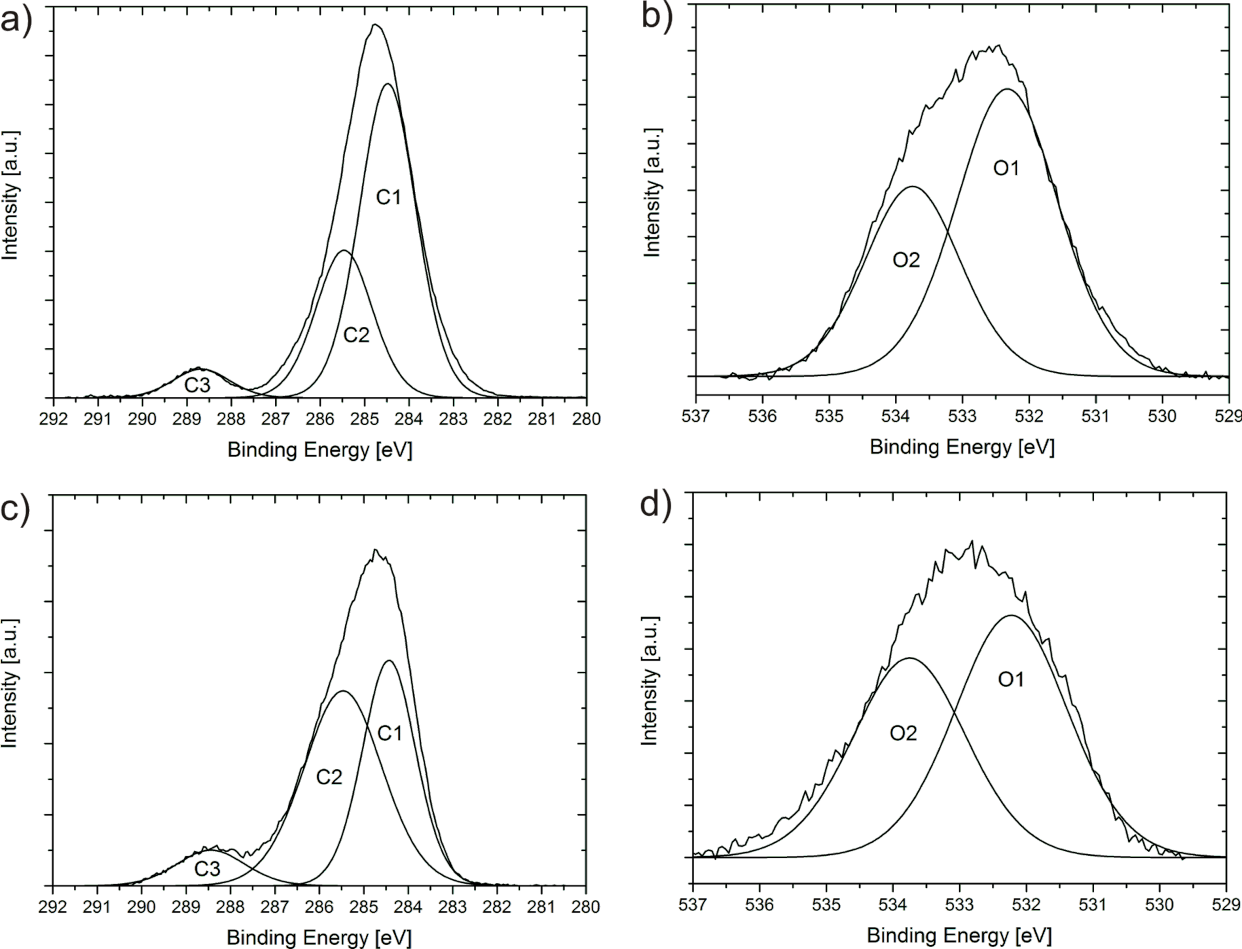
XPS spectra of low-density (a,b) and high-density (c,d) carbon nanofoams showing the C1s (a,c) and O1s (b,d) spectral distributions for low- and high-density foams, respectively.

In Figure 3, the XPS spectra of low- and high-density carbon nanofoams are shown. The deconvolution of the C1s peaks leads to the C1 and C2 peaks which are assigned to sp^2^- and sp^3^-type carbons, corresponding to electron binding energies of 284.4 and 285.4 eV, respectively [54]. An additional peak, C3, is detected at 288.6 and 288.5 eV, for the low- and high-density foams, respectively.

Comparing the C1s distributions of Figure 3a,c for the two carbon foams we find that the shape of the C1s peak significantly changes from low to high density. In the low-density foam (a) the C1s distribution is close to that of the distribution for sp^2^-type carbon with a small asymmetry toward higher binding energies. The distribution is quite narrow with a FWHM of about 2 eV. When deconvoluted, we find the areas of 64% and 30% for sp^2^ and sp^3^ components, respectively. The distribution in Figure 3c however shows a remarkably different sp^2^/sp^3^ ratio, with 40% and 51% for sp^2^ and sp^3^ components, respectively. For the high-density sample, the sp^3^-content gives a strong contribution, which suggests that diamond-like regions are present.

There is an additional distribution found at about 288.5 eV which is due to various types of carbon oxides [55–58]. This peak increases from 5.9% to 8.2% for low- to high-density foams and becomes broader. This suggests that more oxygen is adsorbed in the high-density foam, and a wider variety of carbon oxide groups are present [55,59].

The O1s spectral distribution (Figure 3b,d) is also different for the two types of foams. Two-curve deconvolution for the low-density foam yields an O1 peak at 532.3 eV and an O2 peak at 533.8 eV, with corresponding relative areas of 62% and 38%. We assign these peaks to C=O and C–O bonded carbon atoms, respectively [60–62]. For the high-density foam, the O1 peak is at 532.2 eV, with 55% relative area, a small downshift from the low-density O1 peak. A significant difference between the two foams is the relative area of the O2 peak, located at 533.8 eV, which is 45% for the high-density foam. We conclude that from low- to high-density foam, the number of C=O bonded carbon atoms is reduced, and there are an increased number of C–O bonded carbon atoms in the high-density foams.

## Discussion

We have demonstrated that the simultaneous existence of high pressure and high temperature in an autoclave with an aqueous solution of sucrose/naphthalene precursor leads to the formation of high-quality carbon nanofoams. The foams have uniform structure, predominantly composed of carbon microspheres. We note that our production is performed at relatively low temperatures of 150–185 °C and without a catalyst. The results show that our method leads to different carbonaceous products if the processing conditions are varied. Compared to other techniques, the hydrothermal method provides us with a mild, green, and fast technique which can easily be scaled up as a low-cost production route for large-scale applications.

In addition, the hydrothermal technique leads directly to carbon nanofoam with no further treatment necessary. This is important since nanocarbon materials often need to be purified after synthesis. For example, production methods such as arc discharge [63] and laser ablation [64] may lead to carbon soot byproducts, and additional purification is required. The nanofoam of our studies has a consistently homogeneous morphology and no such post-treatment is necessary to obtain a good-quality product.

From the HIM images we see that the carbon nanofoam consists of a conglomeration of spherical bodies. The microspheres generally have a narrow size distribution. An attractive interaction keeps the spheres together, forming the overall foam structure. We find that if other variables are kept constant, the size of the spheres is dependent on the process temperature. At higher temperatures we still find spherical micropearls but with increased diameters. This suggests that these species form by successive addition of layers added to a spherical core. Growth may occur in a concentric way leading to larger diameter spheres with increasing process temperature. In addition, a higher process temperature accelerates the growth process, facilitates the development of higher structural order and leads to a more advanced stage of carbonization. In the high-density foams, the micropearls tend to be connected to each other, with different neck-size diameters between adjacent pearls. Dangling bonds on the surface of the micropearls may lead to high surface reactivity and consequently to the aggregation of the spheres. In fact, such effects have been observed in the growth of carbon structures when the residence time in the autoclave was extended [65].

The advanced growth of the micropearls seems to be accompanied with a thickening of the walls of the graphitic scaffold. We assume that the wall structure starts with single- to few-layer graphene at the beginning of the carbonization process. Then, with further growth, additional layers of graphene are added to the wall structure. Therefore, the foams are light weight when formed in the initial stage of formation. In the experiment, this is done by using low-temperature in the hydrothermal process. At higher temperature, however, further progression of growth occurs, leading to a thickening of the wall structure and an increase in mass density. We found that the temperature used for the production of the low-density foams is close to the lower limit where foams start to form. This observation supports our assumption that there is a successive wall thickening with increasing temperature in the autoclave.

Our experiments suggest that the micropearls have an internal structure made of thin graphitic walls, which change from single-layer to multi-layer graphene with increasing progression of growth. A strictly two-dimensional graphene wall structure however does not explain the spherical shape of the micropearls. Therefore, we assume that the nanofoam scaffold rather consists of curved graphene layers. In addition, these layers need to have a certain degree of disorder providing the porous structure of the pearls. The relatively broad Raman G and D peaks indicate that in-plane and out-of-plane Raman excitations are present.

While Raman spectroscopy probes the bulk of a sample, the information provided by XPS measurements is strongly surface related. The small (<10 nm) inelastic mean free path of the ejected photoelectrons is the crucial factor determining the surface sensitivity of XPS, which enables the quantitative estimation of the elemental composition and the chemical environment in the direct vicinity of the sample surface [66].

The XPS spectra in Figure 3 show an increased sp^3^ contribution for higher-temperature-grown samples. We suggest that this originates from the neck areas between the pearls. In the neck section, the lattice structure is probably quite different from the structure in the sphere. The spherical morphology can be due to the presence of a 5/6 network with isolated pentagons surrounded by hexagons, similar to the fullerene structure. However, this structure cannot be used for the negative-curvature surfaces in the neck area. Heptagons or sp^3^-related units need to be incorporated for obtaining these areas connecting the microspheres. This may lead to the observed increase of sp^3^-character when the micropearls are connected.

## Conclusion

In this study we have shown that the hydrothermal processing of sucrose with the addition of a small amount of naphthalene results in the formation of carbon nanofoam. The morphology of the obtained foam depends on the process temperature in the hydrothermal carbonization. High-quality foams with uniform structures are produced using a green method with a nontoxic precursor material. Foams with different mass densities can be produced by variation of the process temperature. Mircopearls are the basic units in the low-temperature foams, while connected pearls form the high-density foams. The transition from low- to high-density foams is accompanied by a change in the graphitization degree as well as a change in the surface electronic configuration. With higher autoclave temperature, the carbonization process becomes more effective, which leads to an increase in sphere size and a thickening of the internal wall structure.

The surface sensitivity of XPS exhibits a distinct difference in the sp^3^ binding contribution of the two types of foams. While higher-density foams show an increased degree of graphitization (as observed with the Raman spectra), they also reveal a significant increase of sp^3^ bonded atoms (as observed with the XPS spectra). We relate this to the surface area network which appears to be very different for the two types of foams. The formation of neck areas between the partially fused micropearls requires the inclusion of heptagons and sp^3^-type connections.

We conclude that the nanofoam materials exhibit physical properties originating from a mixture of carbon atoms with sp^2^ and sp^3^ hybridization. The structure in the core of the micropearls is based on a mostly sp^2^-type, three-dimensional network, whose specific property stems from the curvature and interconnection of graphitic basal planes. We assume that the low-density carbon nanofoam has the structure of such a three-dimensional interconnected network of graphene. Depending on the growth conditions, the sp^2^ carbon atoms can also form pentagonal and heptagonal rings with sp^3^-type interconnections built into the hexagonal network. The results show that the combination of various types of n-fold rings and hybrid sp^2^–sp^3^ structures makes carbon unique among the chemical elements, and it can lead to the formation of a variety of geometrical carbon nanofoam structures.
